# Molecular Mechanism of the Regulatory Effect of Schisandrol A on the Immune Function of Mice Based on a Transcription Factor Regulatory Network

**DOI:** 10.3389/fphar.2021.785353

**Published:** 2021-12-20

**Authors:** Guangyu Xu, Yanbo Feng, Han Li, Cong Chen, He Li, Chunmei Wang, Jianguang Chen, Jinghui Sun

**Affiliations:** College of Pharmacy, Beihua University, Jilin, China

**Keywords:** schizandrol A, network regulation, transcription factor, immune function, microarray

## Abstract

The molecular mechanism of the regulatory effed of schizandrol A (SA) on the immune function of cyclophosphamide-induced immunosuppressive mice was explored in this study. On the basis of 1619 differentially expressed genes related to the regulatory effect of SA on the immune function of mice screened in our previous study, transcription factors and their corresponding target genes were screened in the Transcriptional Regulatory Element Database (TRED), and a transcription factor target gene regulatory network was constructed. The key nodes of the network were statistically analyzed to clarify the role of transcription factors in the regulatory network. The correlation of network genes with diseases was analyzed with an online annotation tool through the Database for Annotation, Visualization and Integrated Discovery (DAVID). Finally, the key factors related to the regulatory effect of SA on the immune function of mice were screened and verified by animal experiments and the detection of related protein expression by western blot analysis. The results showed that SA could alleviate the immunosuppression induced by cyclophosphamide in mice and regulate the protein expression of Jun, Trp53, and Creb1 in the spleen tissue of mice, together with the transcription factors Atf4 and E2f2. SA may thus play a role in the alleviation of some immunity-related diseases (such as cancer) by regulating the immune function of mice through multiple genes and their transcription factors.

## Introduction

The immune system, composed of immune organs, immune cells, and independent lymphatic vessels, not only plays a defense function but also serves as a main communication system among tissues, organs, and organic systems in the body. Due to the close correlation of immune system function with human health; infections, inflammatory diseases, and even cancers will easily occur if the immune system is abnormal. At present, the pathogenesis of immune system abnormalities remains unclear, and chemical synthetic drugs, human or animal immune products, and microbial drugs are mainly used for the treatment of abnormalities in the clinic, but these drugs have different degrees of side effects (Peng et al., 2020; Vavla et al., 2020). Therefore, researchers have begun to look for immunomodulatory substances from safer natural products and their extracts.

*Schisandra chinensis*, the dried mature fruit of *Schisandra chinensis* (Turcz.) Baill, is a traditional Chinese medicine in China. *Schisandra chinensis* is listed as the top-grade medicine in “The Shen Nong Ben Cao Jing” (the first existing traditional Chinese medicine classic); it has been used for more than 2000 years and is a famous traditional Chinese medicine for the effect of “Yi Qi” (tonifying Qi, improving body function and increasing the body’s resistance to external pathogenic factors) (Xing et al., 2021). The main components of *Schisandra chinensis* are lignans, and one of the most active components in lignans is schizandrol A (SA) (Arken, 2019; Liu et al., 2019; Song et al., 2020). Modern pharmacological studies show that SA may enhance the phagocytosis of macrophages and promote the formation of antibodies to regulate immunity (Xu et al., 2018a), but the information pathway and related key regulatory genes have not been deeply studied, although immunoregulation experiments at the animal level have been performed in many related studies. It has been found that the regulation of gene expression is a very complex process, and transcription factors are a group of protein molecules that can specifically bind to a specific sequence upstream of the 5′ end of a gene to ensure that the target gene is expressed at a specific intensity in a given time and space (Papavassiliou and Papavassiliou, 2016). The expression of mRNA is regulated by a variety of factors, especially transcription factors, and the role of transcription factors in the regulation of mRNA expression and its mechanism can be directly and better understood through the construction of a regulatory network of transcription factors and their target genes (Arora et al., 2013).

We completed a mRNA expression profile experiment on the regulatory effect of SA on the immune function of mice and screened 1619 differential genes related to immune function (Xu et al., 2018a). In this study, transcription factors were screened through the Transcriptional Regulatory Element Database (TRED), a regulatory network of transcription factors and their target genes was constructed, and the key regulatory factors were analyzed through the central nodes of the regulatory network and verified by animal experiments and western blot analysis. These findings are expected to elaborate the molecular mechanism of the regulatory effect of SA on the expression of differential genes involved in the immune function of mice to provide a theoretical basis for the further application of *Schisandra chinensis*.

## Materials and Methods

### Chemicals and Reagents

Schisandrol A (Preferred Biotechnology Co., Ltd., > 98%, Chengdu, China, No. 19110503); Cyclophosphamide (Cy) for Injection (Baxter Oncology GmbH, Halle, Germany, H20160467); Concanavalin A (Con A, type IV; Sigma-Aldrich, Merck KGaA, Darmstadt, Germany); polyclonal antibodies Jun, Trp53, and Creb1 (ABclonal, Wuhan, China; No. 3560496003, 3560238002, and 0003310201); electrogenerated chemiluminescence (ECL) color liquid (Biyuntian Biological Products Co., Ltd., Shanghai, China, No. 9966444330).

### Animal Grouping and Administration

A total of 180 male ICR mice (Changchun Yisi Experimental Animal Technology Co., Ltd., Changchun, China) weighing 18–22 g were raised in an animal room in a 12 h day/night cycle. The room was disinfected once a week, and the bedding for the mice was changed once a week. The temperature was controlled at 20–24°C, and the humidity was approximately 45–50%. The animal experiment was approved by the Institutional Animal Care and Use Committee (IACUC) of Beihua University, and all the experimental procedures were carried out in accordance with the “Regulations on the Administration of Experimental Animals” approved by the State Council of the People’s Republic of China.

**FIGURE 1:**
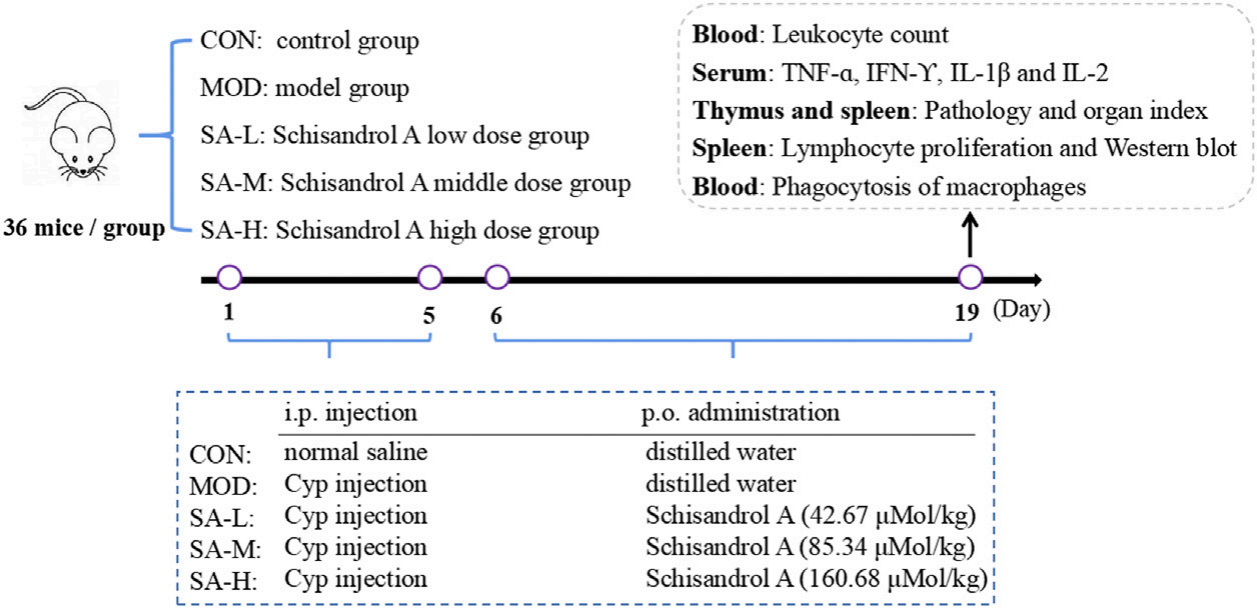
Experimental protocol.

The mice were randomly divided into five groups: the control group, model group, low-dose SA group (SA-L, 42.67 μMol/kg SA), medium-dose SA group (SA-M, 85.34 μMol/kg SA), and high-dose SA group (SA-H, 160.68 μMol/kg SA), with 36 mice in each group. Mice in the model and SA-treated groups were given 40 mg/kg cyclophosphamide once a day by intraperitoneal injection for 5 days, and those in the control group were given an equal volume of normal saline in the same manner. On the next day, mice in the SA-treated groups were given the corresponding doses of SA solution by gavage, and those in the control group and model group were given an equal volume of distilled water by gavage for 14 days. After the last intragastric administration, all the mice fasted for 12 h prior to the collection of the samples. The specific experimental protocol is shown in Figure 1.

### Measurement of Leukocyte Count, TNF-α, IFN-γ, IL-1β, and IL-2 Levels and Organ Index

Twelve mice from each group were anesthetized by intraperitoneal injection of 4% chloral hydrate (0.10 mL/10 g). Then, the eyeballs were removed to collect blood samples from the mice. Twenty microliters of the blood of mice was mixed with acetic acid, and the number of white blood cells in the blood was counted with a counting plate under a microscope. The rest of the blood was centrifuged (3000 r/min, 10 min) to obtain the serum, and the levels of TNF-α, IFN-γ, IL-1β, and IL-2 in the serum were detected by ELISA. The thymus and spleen of mice were removed after the collection of blood samples, washed with normal saline, and then weighed. The thymus and spleen indices were calculated according to the following formula, and the spleen samples were stored at −80°C for subsequent experiments.

Thymus index=thymus weight/body weight (g) × 100%

Spleen index=spleen weight/body weight (g) × 100%

### Observation of the Proliferation of Spleen Lymphocytes

Another 12 mice from each group were intraperitoneally injected with 4% chloral hydrate (0.10 mL/10 g) for anesthesia, and then the spleen of mice was carefully removed on a sterile table and ground into pieces. The ground spleen tissue was added to 4 mL of erythrocyte lysis buffer to prepare a lymphocyte suspension, the lymphocyte suspension was centrifuged (15000 r/min, 10 min, 4°C), and the supernatant was discarded. The residual cells were added to fetal bovine serum (FBS) and 2.5 mL of culture medium, which was mixed by repeated pipetting. Then, the mixture was drawn with a pipette, trypan blue was added for dyeing, and 1 minute later, 20 μL of the mixture was added onto a counting plate for cell counting. The cells were seeded onto 24-well plates at a density of 1 × 10^6^ cells/mL, and 50 μL of Con A was added to each well. Then, the plates were placed in a CO_2_ incubator at 37°C for 3 days for the culture of cells, and the proliferation of cells was evaluated by MTT assays.

### Observations on Macrophage Phagocytosis

Another 12 mice from each group were injected with 50% India ink and normal saline mixture *via* the tail vein, and 4% chloral hydrate (0.10 mL/10 g) was intraperitoneally injected for anesthesia. At 4 and 12 min after the injection of India ink, 20 μL of the blood was taken from the inner canthus of mice and then immediately injected into sodium carbonate solution, and the optical absorbance (OD) of the solution at 675 nm was measured by a microplate reader. Then, the liver and spleen were weighed. The phagocytosis of mouse macrophages was expressed by phagocytic index α, which was calculated as follows:

$$K=\left(\log A_{4}\;-\log A_{12}\right)/\left(t_{12}-t_{4}\right)$$

Phagocytic index α = body weight/liver weight + spleen weight

$$\times k^{\mathrm{1/3}}$$

where *A*_4_ and *A*_12_ represent the absorbance values at 4 and 12 min, respectively, and *t*_12_ and *t*_4_ represent the 12th minute and the 4th minute, respectively.

### Detection of Jun, Trp53, and Creb1 by Western Blot

The spleen tissue (90 mg) was cut into pieces, and then the spleen tissue pieces were mixed with lysis buffer (810 μL) in a tube for the preparation of the spleen tissue homogenate. The cells were lysed by putting the tube on ice for 1 h. The homogenate was centrifuged at 12000 rpm and 4°C for 10 min, and the supernatant was obtained. The butylethanoacrylate (BCA) method was used to determine the concentration of proteins in the supernatant, and the proteins were denatured by boiling the protein sample for 5–10 min. A 10% SDS-polyacrylamide gel was used to separate the proteins, before they were transferred onto PVDF membranes. Five percent skim milk was used for blocking for 2 h, and then the membranes were incubated with primary antibodies against Jun, Trp53, and Creb1 at 4°C overnight. Then, the membrane was washed three times with TBST for 10 min each time and incubated with the corresponding secondary antibodies for 1 h at room temperature. Finally, the proteins were visualized with ECL developer, and their grayscales were analyzed using a Champchemi professional + automatic multicolor fluorescence and chemistry gel imaging system (Saizhi Technology Co., Ltd., Beijing, China), in which the ratio of target protein and GAPDH was used to represent the relative expression of the proteins.

### Acquisition of Chip Data

All chip data in this study were obtained through GEO DataSets of the National Center for Biotechnology Information (NCBI) database, and the GES accession was GSE97316 (Xu et al., 2018a).

### TF mRNA Gene Network Construction

The related transcription factors (TFs) and their target genes were obtained according to the mRNA expression profiles after the analysis of chip data and through searching in the Transcriptional Regulatory Element Database (TRED), and their network was constructed by applying Cytoscape software. In the TF-gene network, the transcription factors are represented as yellow rhombi, the target genes are represented as blue circles, and dotted lines with arrows from the source to the targets are used to connect the TFs and their target genes.

### Gene Function Annotation Analysis

The differential gene function analysis and pathway classification were conducted through the Database for Annotation, Visualization and Integrated Discovery (DAVID) and Kyoto Encyclopedia of Genes and Genomes (KEGG), respectively.

### Statistical Analysis

Analysis of variance (ANOVA) was used for the analysis of data, followed by Dunnett’s multiple comparison testing, in which the data are expressed as the mean±*SD* (standard deviation), and a value of *p* < 0.05 is considered to indicate a difference with statistical significance.

## Results

### Microarray Data of mRNA Expression Profiles

**FIGURE 2:**
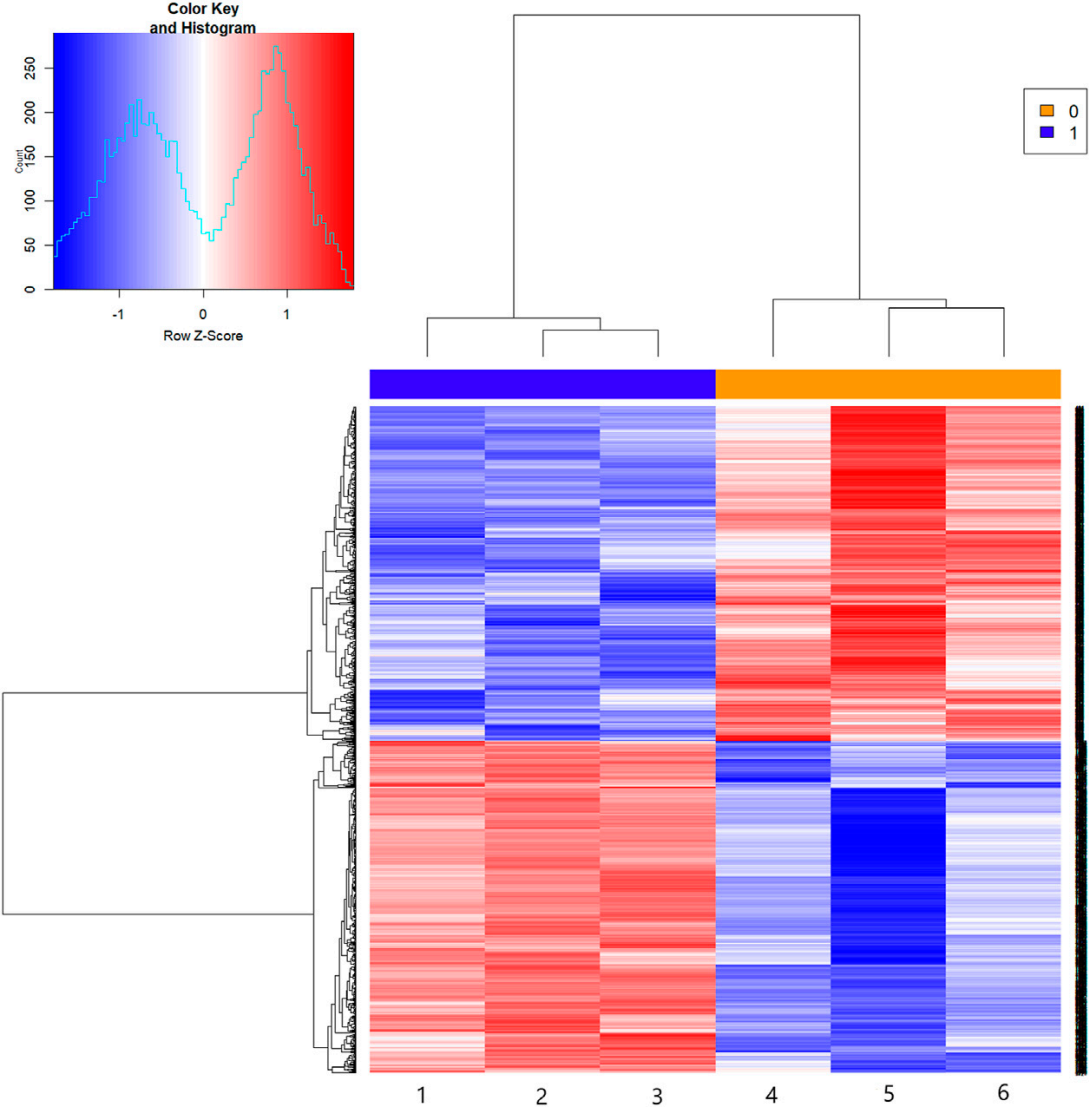
The hot figure of mRNA expression profile microarray. Notes: Group 1-3: the model group; group 4-6: SA-H group. Red parts indicate up-regulation, blue parts indicate down-regulation.

**FIGURE 3:**
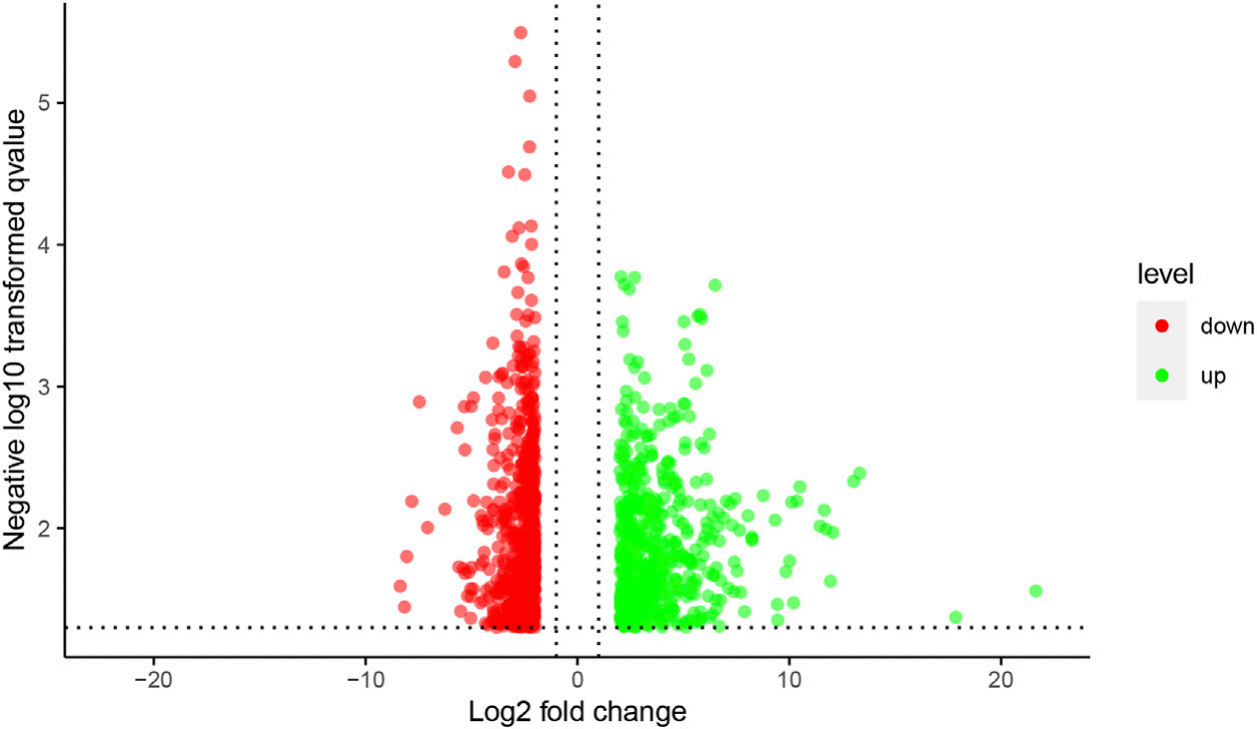
The volcano figure of mRNA expression profile microarray. Notes: Red dots indicate up-regulation genes, green dots indicate down-regulation genes.

The results of the mRNA microarray (Figure 2 and Figure 3) showed that in contrast to the model group (MOD group), there were 1619 differentially expressed genes in the SA-H group, of which 813 (50.22%) were significantly upregulated and 806 (49.78%) were significantly downregulated.

### Regulatory Network of Transcription Factors and Their Target Genes Related to the Regulatory Effect of SA on the Immune Function of Mice

The predictive analysis of the potential transcription factors in 1619 genes through the Translational Regulatory Element Database (TRDE, https://cb.utdallas.edu/cgi-bin/TRED/tred.cgi?process=home) showed that there were six transcription factors and 257 corresponding target genes (Table 1).

**TABLE 1:** Transcription factors and their corresponding target genes.

TF	TG No	Description	Gene ID
Atf4	107	Mus musculus activating transcription Factor 4 (Atf4), transcript variant 1, mRNA [NM_009716]	11911
E2f2	80	Mus musculus E2F transcription Factor 2 (E2f2), mRNA [NM_177,733]	242705
Nfix	28	Mus musculus nuclear Factor I/X (Nfix), transcript variant 2, mRNA [NM_010906]	18032
Trp63	25	Mus musculus transformation related protein 63 (Trp63), transcript variant 3, mRNA [NM_001127261]	22061
Hif3a	13	Mus musculus hypoxia inducible Factor 3, alpha subunit (Hif3a), transcript variant 2, mRNA [NM_016868]	53417
Esr2	4	Mus musculus estrogen receptor 2 (beta) (Esr2), transcript variant 1, mRNA [NM_207,707]	13983

Note: TG, target gene; TF, transcription factor.

**FIGURE 4:**
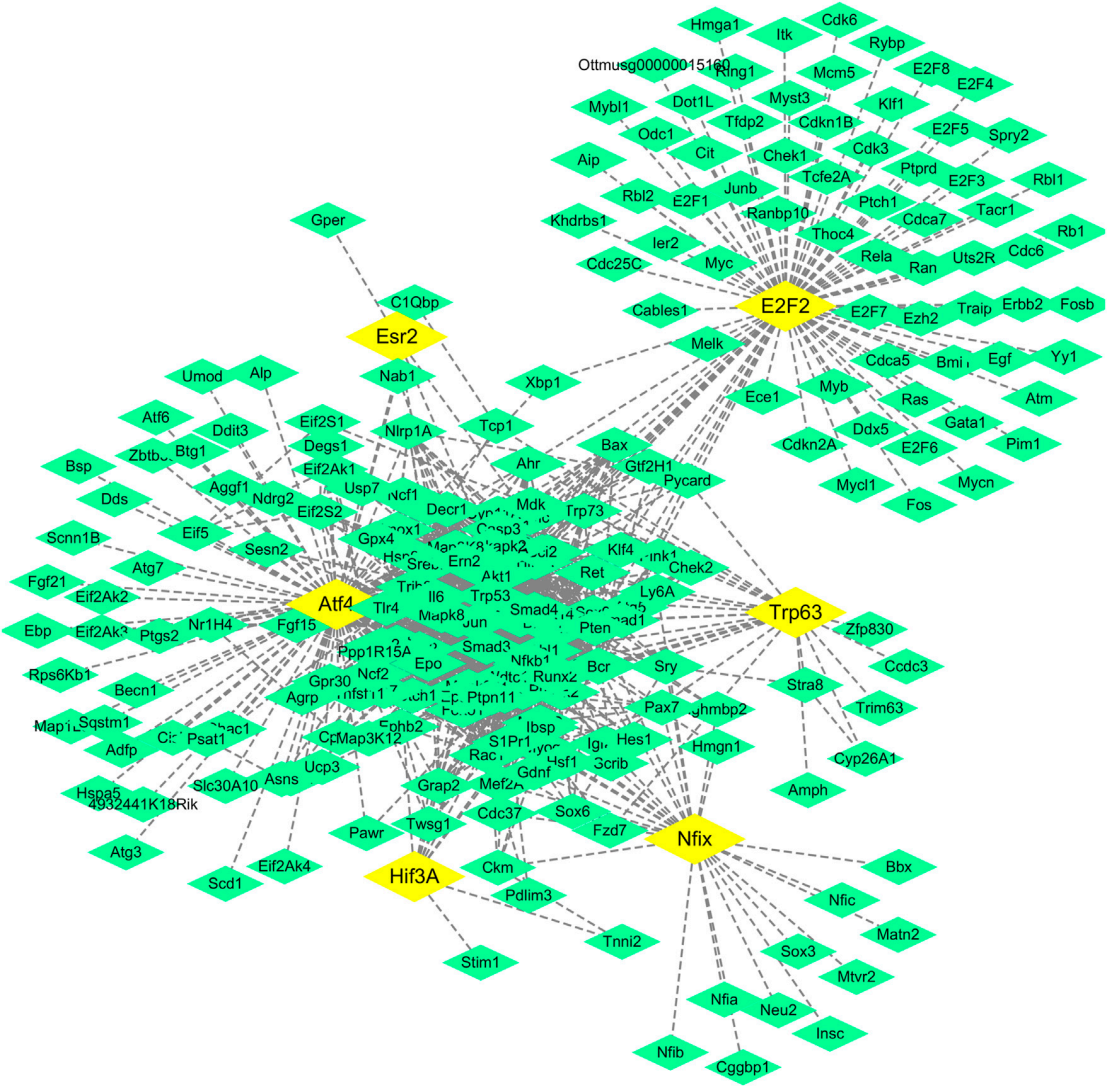
Regulatory network of transcription factors and their target genes. Notes:The yellow boxes are transcription factors, the green boxes are target genes.

In addition, Cytoscape software was used to construct a regulatory network of the six transcription factors and 257 corresponding target genes (Figure 4), and the regulatory network showed that there were 18 target genes regulated by more than two transcription factors and six target genes regulated by three transcription factors (Table 2).

**TABLE 2:** Statistics of genes regulated by transcription factors.

TG regulated by TF	No. of TF regulating TG	Regulatory TF	Node
Akt1	3	Atf4 Trp63 E2f2	78
Trp53	3	Atf4 Trp63 E2f2	71
Creb1	3	Atf4 Hif3A E2f2	57
Casp3	3	E2f2 Atf4	53
Pten	3	Atf4 Hif3A E2f2	51
Cdkn1A	3	Trp63 E2f2 Nfix	48
E2F2	2	Trp63 E2f2	80
Bcl2	2	E2f2 Atf4	54
Wdtc1	2	Trp63 Atf4	32
Arnt	2	Hif3A E2f2	20
Ret	2	E2f2 Atf4	19
Il3	2	Esr2 Atf4	15
Pomc	2	Esr2 Atf4	15
Trp73	2	Trp63 E2f2	14
Grap2	2	Grap2 Atf4	13
Klf4	2	E2f2 Atf4	12
Pycard	2	Trp63 E2f2	6
Xbp1	2	E2f2 Atf4	2

Note: TG, target gene; TF, transcription factor.

### Statistical Analysis of Transcription Factor Network Nodes

The network nodes in the regulatory network of transcription factors were counted. There were 19 genes with more than 40 nodes, of which the top three genes with the most nodes were Atf4 (107 nodes), E2f2 (80 nodes), and Akt1 (78 nodes) (Table 3); moreover, Akt1 and E2F2 were also genes regulated by most transcription factors.

**TABLE 3:** Statistics of network nodes.

Gene	Node	No. of TF regulating TG
Atf4	107	1
E2f2	80	1
Akt1	78	3
Jun	76	1
Trp53	71	3
Mapk14	69	1
Mapk3	68	1
Mapk8	63	1
Il6	63	1
Creb1	57	3
Bcl2	54	2
Casp3	53	2
Pten	51	3
Esr1	50	1
Nfkb1	49	1
Vegfa	48	1
Cdkn1A	48	3
Rhoa	42	1
Foxo1	41	1

Note: TG, target gene; TF, transcription factor.

### GO Functional Annotation Analysis of Differential Genes Related to the Regulatory Effect of SA on Immune Function in Mice

The GO signaling pathway function annotation of the 1619 differential genes related to the regulatory effect of SA on the immune function of mice was conducted, and the top 10 pathways were selected according to their *p* values (Table 4), including one signaling pathway (HIF-1 signaling pathway), four cancer-related pathways (Pathways in cancer, Pancreatic cancer, MicroRNAs in cancer and HIF-1 signaling pathway) and three virus-related pathways. The HIF-1 signaling pathway is most related to this immunosuppressive model in GO terms. Hepatitis B, HTLV-I infection, and prostate cancer are related to transcription factors (Atf4 and E2f2). Pathways in cancer and viral carcinogenesis contain differential gene expression (Jun, Trp53, and Creb1).

**TABLE 4:** GO functional annotation analysis.

Pathway	Gene No	*p* Value	Benjamini
Pathways in cancer	45	2.0E-20	3.6E-18
Hepatitis B	26	9.3E-16	8.1E-14
Chronic myeloid leukemia	19	1.6E-14	1.0E-12
Cell cycle	23	2.0E-14	9.3E-13
Pancreatic cancer	18	3.4E-14	1.2E-12
HTLV-I infection	30	2.2E-13	6.6E-12
Prostate cancer	18	5.0E-12	1.3E-10
MicroRNAs in cancer	27	5.9E-11	1.4E-9
HIF-1 signaling pathway	17	1.6E-10	3.3E-9
Viral carcinogenesis	23	3.5E-10	6.4E-9

### Effects of SA on Serum TNF-α, IFN-γ, IL-1β, and IL-2 Levels and Organ Indices

**FIGURE 5:**
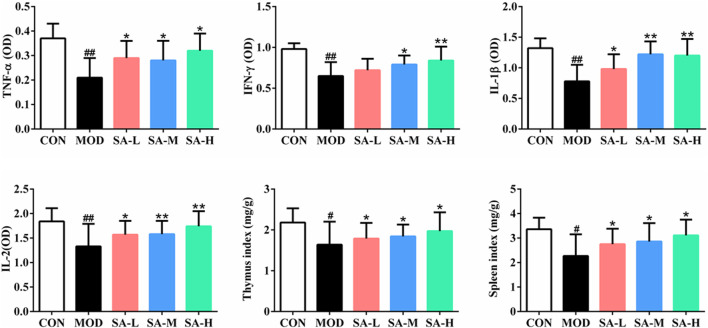
Effects of SA on the serum TNF-α, IFN-γ, IL-1β and IL-2 levels and the organ indexes. Notes: Groups: CON, control group; MOD, model group; SA-L, Schisandrol A low-dose group; SA-M, Schisandrol A medium-dose group; SA-H, Schisandrol A high-dose group. All the values were expressed as mean ± standard deviation (*n* = 12). #: *p* < 0.05, *vs*. CON; ##: *p* < 0.01, *vs.* CON; *: *p* < 0.05, *vs*. MOD; **: *p* < 0.01, *vs*. MOD.

As shown in Figure 5, compared with those in the control group, the number of white blood cells, TNF-α, IFN-γ, IL-1β, and IL-2 levels in the serum of mice, and spleen index and thymus index of mice were significantly decreased in the model group (*p* < 0.05, *p* < 0.01); compared with those in the model group, the number of white blood cells, TNF-α, IFN-γ, IL-1β, and IL-2 levels in the serum of mice and spleen index and thymus index of mice were increased in varying degrees in SA groups (*p* < 0.05, *p* < 0.01).

### Effects of SA on the Leukocyte Number, Lymphocyte Proliferation, and Macrophage Phagocytosis of Mice

**FIGURE 6:**
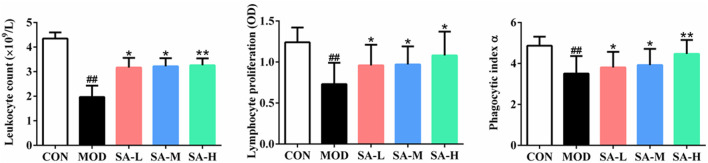
Effects of SA on the leukocyte number, lymphocyte proliferation and macrophage phagocytosis of mice. Notes: Groups: CON, control group; MOD, model group; SA-L, Schisandrol A low-dose group; SA-M, Schisandrol A medium-dose group; SA-H, Schisandrol A high-dose group. All the values were expressed as mean ± standard deviation (*n* = 12). ##: *p* < 0.01, *vs*. CON; *: *p* < 0.05, *vs*. MOD; **: *p* < 0.01, *vs*. MOD.

As shown in Figure 6, compared with those in the control group, the leukocyte number, the proliferation of lymphocytes, and the phagocytosis of macrophages of mice in the model group were significantly decreased (*p* < 0.05, *p* < 0.01), and compared with those in the model group, the number of leukocytes, the proliferation of lymphocytes, and the phagocytosis of macrophages of mice in SA group were significantly increased (*p* < 0.05, *p* < 0.01).

### Effects of SA on the Expression of Jun, Trp53, and Creb1 Proteins

**FIGURE 7:**
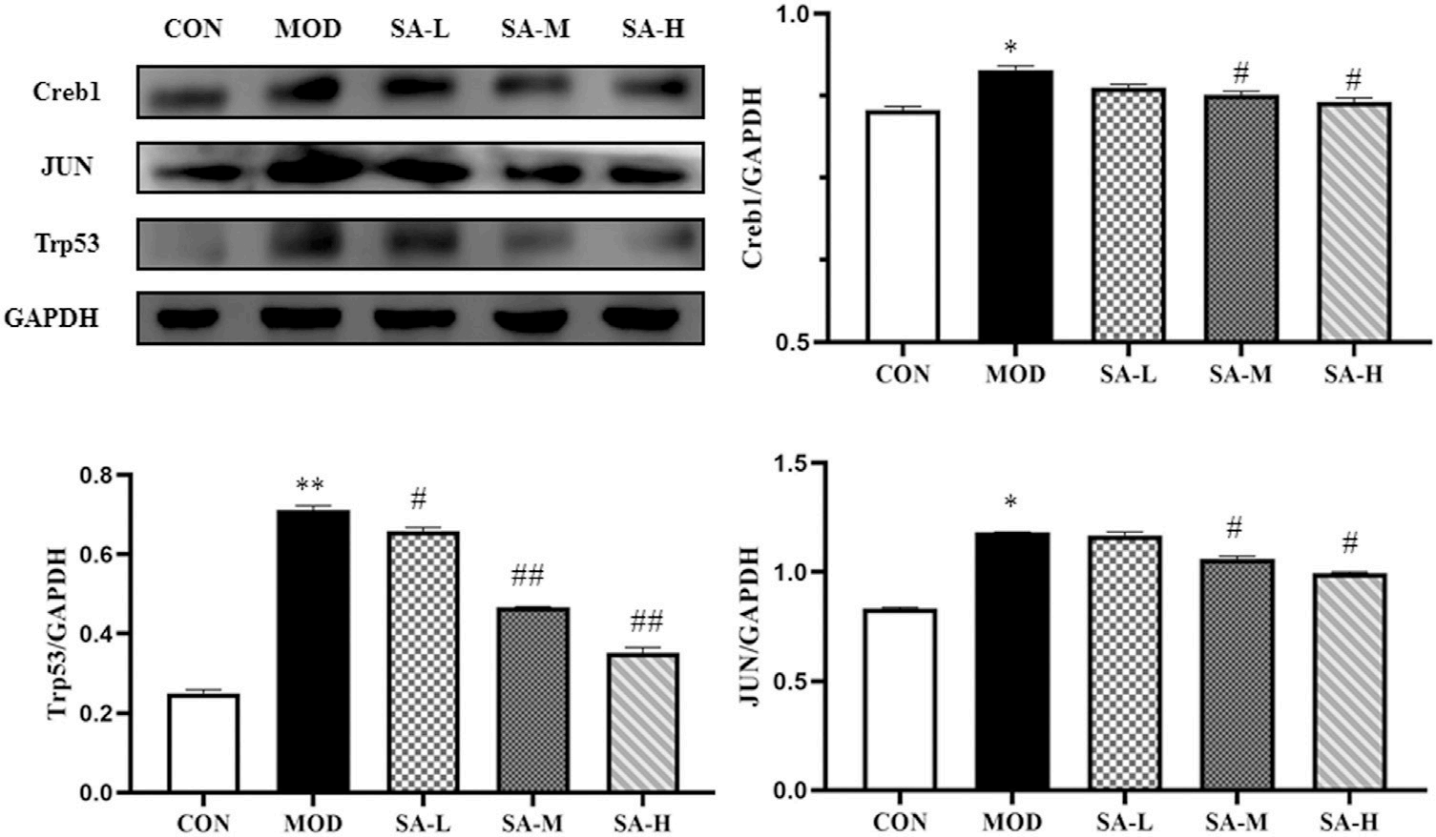
Effects of SA on the expression of Jun, Trp53 and Creb1 proteins. Notes: Groups: CON, control group; MOD, model group; SA-L, Schisandrol A low-dose group; SA-M, Schisandrol A medium-dose group; SA-H, Schisandrol A high-dose group. All the values were expressed as mean ± standard deviation (*n* = 3). *: *p* < 0.05, *vs*. CON; **: *p* < 0.01, *vs*. CON; #: *p* < 0.05, *vs*. MOD; ##: *p* < 0.01, *vs*. MOD.

A regulatory network of transcription factor genes and their corresponding targets was constructed (Figure 4), in which Jun, Trp53, and Creb1 were found to be the key nodes. If the verification results of these three nodes were consistent with the prediction results, the correctness of the regulatory network construction could be proven, so we used western blot analysis to verify the protein expression of these three genes. As shown in Figure 7, the expression of Jun, Trp53, and Creb1 proteins in the spleen tissue of mice in the model group was significantly higher than that in the control group (*p* < 0.01), and the expression of the Jun, Trp53, and Creb1 proteins in the spleen tissue of mice in the different SA-treated groups was lower than that in the model group to varying degrees (*p* < 0.05, *p* < 0.01).

## Discussion

At present, immune system diseases have become the main diseases endangering human health, and the research and development of immunity-enhancing drugs are becoming increasingly important. In this study, the effect of SA on the immune function of mice and its mechanism were investigated in cyclophosphamide-induced immunosuppressive mice. Cyclophosphamide, a classical immunosuppressant, can reduce the proliferation of lymphocytes and the levels of TNF-α, IFN-γ, IL-1β, and IL-2 to cause immune function disorders. It can be seen from the experimental results of this study that SA can significantly increase the immune organ indices and the number of white blood cells, improve the proliferation of lymphocytes and the phagocytosis of macrophages, and elevate the level of immune factors of immunosuppressive mice induced by cyclophosphamide.

Our team previously completed an mRNA expression profile experiment to examine the regulatory effect of SA on the immune function of mice and screened 1619 differential genes. These 1619 differential genes were also predicted and analyzed through TRDE in this study, of which six transcription factors and their corresponding 257 target genes were found, and a differential gene transcription factor regulatory network related to the regulatory effect of SA on the immune function of mice was established (Figure 2). A series of statistical analyses on the regulatory network of transcription factors was performed, and it was found that many of the key factors were closely related to the regulation of immune system function.

The results showed that there were six transcription factors in the regulatory network of transcription factors (Figure 4), among which Atf4 (corresponding to 107 target genes) and E2f2 (corresponding to 80 target genes) were the transcription factors with the most corresponding target genes, and the number of corresponding target genes of the other transcription factors was less than 80.

Atf4 (activating transcription Factor 4) is a fast response gene in the early stage of stress, and its expression is closely related to inflammation, homeostasis, and tumorigenesis. In a variety of inflammatory models, Atf4 plays a brake role in the inflammatory response, and Atf4 is abundantly expressed in macrophages, participating in the regulation of HDL on the TLR-mediated inflammatory response (Ameri and Harris, 2008). E2f2 (E2f transcription Factor 2) is a member of the E2F family, and the E2F family is an important transcription factor that regulates the cell cycle and apoptosis and is widely involved in the tumorigenesis of various tissues and organs (Indovina et al., 2015). Studies have shown that E2f2, with a strong carcinogenic ability, can maintain the homeostasis of cells together with the P53 protein and play a precise role in regulating the transformation of cells and the progression of the cell cycle, and the imbalance of this homeostasis caused by related factors is one of the mechanisms of tumorigenesis (Li et al., 2016).

Genes regulated by transcription factors (Table 2) in the regulatory network of transcription factor target genes (Figure 4) were analyzed statistically, and 18 target genes regulated by more than two transcription factors were found, including six target genes (Akt1, Trp53, Creb1, Casp3, Pten, and Cdkn1A) regulated by three transcription factors. Among them, a mutation in Akt1 in human breast cancer and gastric cancer has been found by researchers, and the mutation can stimulate this kinase by enhancing its membrane connection, thus directly correlating Akt1 with the occurrence of cancer (Xu et al., 2018b; Zhang et al., 2019; Yehia and Eng, 2020). The Trp53 gene, a well-known cancer gene, is related to the pathogenesis of many cancers (Livon et al., 2019; Yin et al., 2019). The Creb protein expressed by Creb1, an important transcription regulatory protein in eukaryotic cells, can regulate gene transcription by interacting with DNA and other transcription regulators. Intracellular phosphorylated Creb can induce the expression of downstream genes of the cAMP response element and then regulate some biological processes, such as the proliferation and differentiation of cells (Brown et al., 2019). The Casps gene is a susceptible gene of Kawasaki disease (Onouchi et al., 2010), and Kawasaki disease is a self-limited immune disease. Caspase-3 encoded by Casp3 is an effector cysteine protease, and the enzyme is activated by activated proteases of the cysteine protease family to initiate the cleavage of downstream proteins and regulate the apoptosis of cells (Choudhary et al., 2015). The Pten gene, located on chromosome 10q23.3, is composed of nine exons and encodes a protein of 403 amino acids with phosphatase activity, and the PTEN protein can inhibit the occurrence and development of tumors by antagonizing the activity of phosphorylases, such as tyrosine kinases (Erickson et al., 2020). Cdkn1A is a cell cycle-related gene located on chromosome 6, and as a tumor suppressor gene, it is widely and expressed at low levels in tumors (Wisnieski et al., 2017). It can be seen based on the above facts that among the genes regulated by the three transcription factors, four genes are related to cancer (congenital or acquired immunodeficiency prone to malignant tumors), one gene is related to immune diseases, and another gene is an important transcription regulator, so it is believed that these six genes are closely related to the immune system.

The network nodes in the transcription factor target gene regulatory network (Figure 4) were counted, and there were five genes with more than 70 nodes, namely, Atf4, E2f2, Akt1, Jun, and Trp53 (Table 3). The Atf4, E2f2, Akt1, and Trp53 genes are all related to cancer, as discussed above, and the Jun gene is closely related to systemic lupus erythematosus (SLE) since some studies suggest that Jun may be involved in the pathogenesis of SLE (Pu et al., 2015). Therefore, these five genes are closely related to cancer and immunity.

**FIGURE 8:**
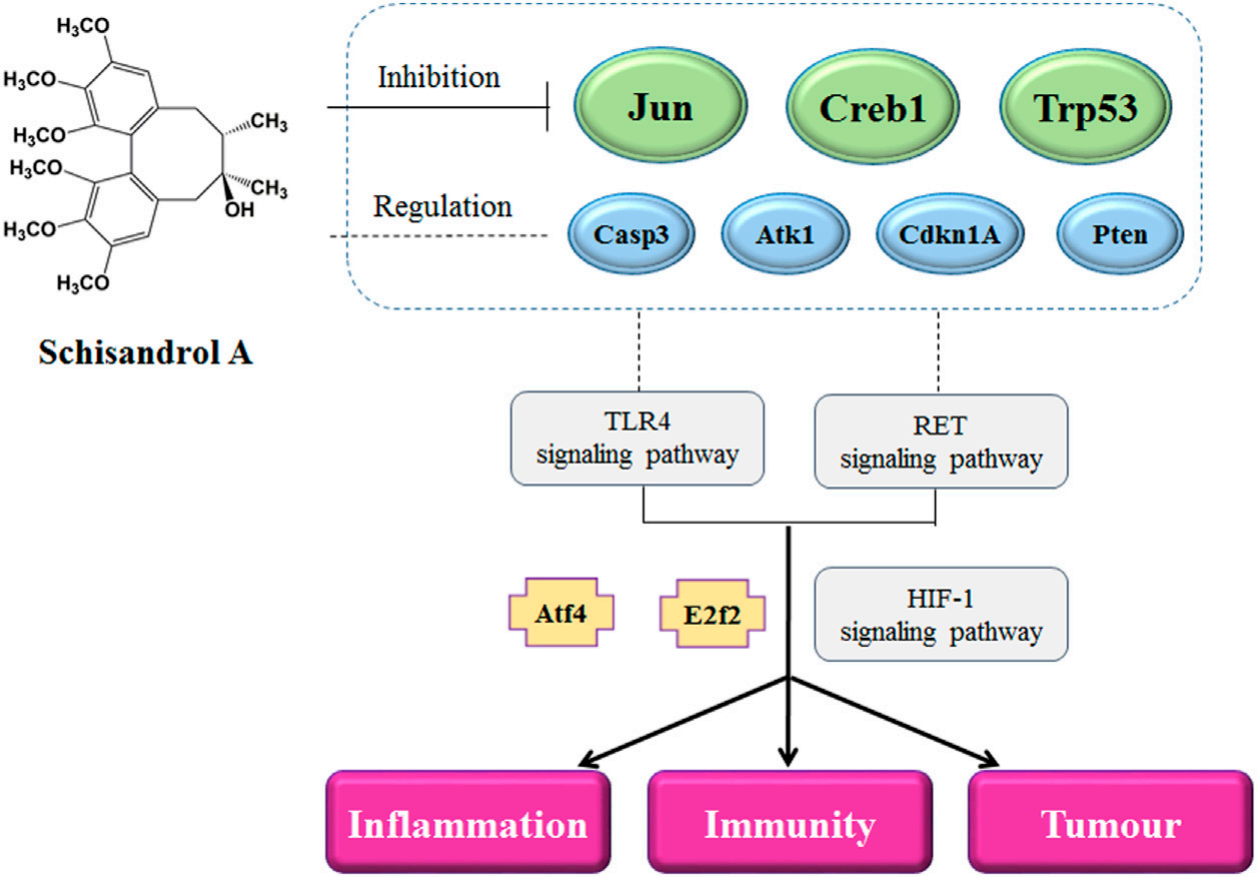
Potential molecular mechanism of the regulation of Schisandrol A on the immune function.

Many diseases are closely related to the immune system, especially cancer. GO functional cluster analysis of the 1619 differentially expressed genes was performed using the DAVID website, and the top 10 pathways with the most significant *p* values were selected, of which four were found to be related to cancer, and one signaling pathway (HIF-1 signaling pathway) was also related to cancer. Some diseases are closely related to immunity-related information pathways, such as HTLV-I infection, viral carcinogenesis, chronic myeloid leukemia, and the cell cycle. At the same time, we also found that most of the central node genes in the transcription regulatory network are related to cancer. Therefore, SA can regulate immunity through other immunity-related disease pathways, and pathway analysis suggests that SA may play a role in cancer-related diseases (Figure 8).

## Conclusion

Six transcription factors and their corresponding 257 target genes were screened out from the 1619 differentially expressed genes related to the regulation of immune function by SA in mice, and a regulatory network of differentially expressed genes related to the regulation of immune function by SA was established. The statistical analysis of the network nodes in the regulatory network of transcription factors and western blot results suggest that jun, Trp53, and Creb1, together with transcription factors Atf4 and E2f2, participate in regulating immune function. SA may play a role in the alleviation of some immune-related diseases by regulating the immune function of mice through multiple genes and their transcription factors.

There are also some limitations in our experiments. For example, we discovered a large number of cancer-related and virus-related pathways in the KEGG pathway clustering, and many key genes are also related to cancer. Although cancer is caused by a decline in the body’s immune system, we should conduct some research on the role of SA in cancer, which is the focus of our future research.

## Data Availability

The datasets presented in this study can be found in online repositories. The names of the repository/repositories and accession number(s) can be found below: https://www.ncbi.nlm.nih.gov/, GSE97316.
